# Associations between social eating contexts and affective states in adolescents: the EHDLA study

**DOI:** 10.3389/fnut.2025.1653965

**Published:** 2025-10-15

**Authors:** José Adrián Montenegro-Espinosa, Fiorella Quiroz-Cárdenas, Rodrigo Yañéz-Sepúlveda, Héctor Gutiérrez-Espinoza, Jorge Olivares-Arancibia, José Francisco López-Gil

**Affiliations:** ^1^School of Medicine, Universidad Espíritu Santo, Samborondón, Ecuador; ^2^Vicerrectoría de Investigación y Postgrado, Universidad de Los Lagos, Osorno, Chile; ^3^Faculty Education and Social Sciences, Universidad Andres Bello, Viña del Mar, Chile; ^4^Faculty of Education, Universidad Autónoma de Chile, Santiago, Chile; ^5^Faculty of Education, School of Physical Education, AFySE Group, Research in Physical Activity and School Health, Universidad de las Américas, Santiago, Chile; ^6^Department of Communication and Education, Universidad Loyola Andalucía, Seville, Spain

**Keywords:** adolescents, family meals, social eating, positive affect, negative affect, Spain

## Abstract

**Introduction:**

Positive and negative affect (PA and NA) are crucial dimensions of emotional experience, that could influence psychological wellbeing in adolescents. Social interactions, particularly around mealtimes, may play a relevant role in adolescent development and wellbeing. This study investigates the associations of family meals and social eating behavior (SEB) with PA and NA in Spanish adolescents.

**Methods:**

This cross-sectional study included 637 adolescents (43% boys) aged 12–17 years from the *Valle de Ricote*, Region of Murcia, Spain. PA and NA were measured using the Spanish version of the Positive and Negative Affect Schedule for Children (PANAS-C10). Family meal frequency was assessed by asking participants how many times they ate with most household members in the previous week. SEB was evaluated with a three-item self-report instrument capturing the frequency and importance of eating with others. Robust generalized linear models (Gaussian family, identity link; “*lmrob*” function, “*robustbase*” package, R) were used to analyze the associations between family meals/SEB and PA and NA, adjusting for age, sex, socioeconomic status, physical activity, sedentary behavior, sleep duration, body mass index, and energy intake.

**Results:**

The median weekly family meals were 14.0 [interquartile range (IQR) 10.0, 16.0], SEB score was 10.0 (IQR 9.0, 11.0), PA score was 19.0 (IQR 15.0, 22.0), and NA score was 8.0 (IQR 6.0, 13.0). Higher SEB scores were related to greater PA [unstandardized beta coefficient (*B*) = 0.44, 95% confidence interval (CI) 0.20 to 0.69, *p* < 0.001]. A borderline association was found between family meals and PA (*B* = 0.09, 95% CI −0.001 to 0.18, *p* = 0.054). Conversely, both higher SEB scores (*B* = −0.21, 95% CI −0.40 to −0.01, *p* = 0.038) and a greater number of family meals (*B* = −0.07, 95% CI −0.14 to −0.001, *p* = 0.048) were associated with lower NA.

**Conclusion:**

Our results suggest a relationship between SEB and PA, while finding no association between family meals and PA, and an inverse relationship between both family meals and SEB with NA. Promoting positive social eating environments and family meals may be associated with adolescents’ emotional wellbeing.

## Introduction

Positive and negative affect (PA and NA) represent the two primary dimensions of emotional experience (1). PA is characterized by high energy, focused attention, and pleasurable engagement with the environment (1) and refers to the internal sense of wellbeing one experiences when a goal is met, a threat is avoided, or one simply feels satisfied with how things stand (2), whereas NA encompasses distressing emotions such as sadness, fear, guilt, and anger (1) and denotes the unpleasant emotions that arise when a task is not completed or is done below the expected standard (2). This division is essential in the study of psychological well-being, as high PA generally relates to adaptive functioning and active interaction with one’s surroundings, whereas elevated NA may indicate susceptibility to emotional disturbances such as symptoms of anxiety and depression (1).

In Spain, the study by Casas and González-Carrasco (2) explored the trajectory of PA and NA during adolescence, using a 5-year longitudinal design with a cohort of 1696 adolescents aged 10–18 years. Research has indicated that PA tends to decrease with age, whereas NA tends to increase (2). Specifically, girls show a more pronounced decrease in PA and a more pronounced increase in NA than boys do (2). Furthermore, PA has a moderate-to-high relationship with life satisfaction, whereas NA has a weaker negative relationship (2, 3). This implies that adolescents with higher PA tend to be more satisfied with their lives, whereas those with higher NA tend to be less satisfied (3). These findings may be associated with the understanding of the role of PA and NA in adolescent subjective wellbeing, offering insights for both theoretical frameworks and practical applications for interventions (4).

In the case of adolescents, one relevant parameter for maintaining adequate wellbeing is the social support of friends, especially the family (5). Since this is a period characterized by the search for external approval, which could play a fundamental role in the wellbeing and development of youth (5). Family support can be observed in various daily actions in which adolescents relate to their family members; one of these dynamics is at mealtimes (6). Family meals can provide a valuable opportunity to establish and/or strengthen family bonds, creating social environments where frequent meals could generate benefits for young people who go beyond good nutrition but rather aim to maintain adequate wellbeing and development, since then constitute a good part of adolescents’ daily lives (7).

Family meals may play a relevant role in adolescents, as do their eating habits, food choices and social and family norms, since they are related to the way in which young people behave and act on a daily basis, thus shaping these behaviors (8). A systematic review (9) revealed that the practice of sharing food in family or social contexts is a significant factor in the psychological wellbeing and integral development of the adolescent population. This review reported that young people who regularly participate in this event tend to have more balanced dietary patterns (9). This not only reduces their chances of suffering from obesity or malnutrition but also has a positive effect on their physical and psychological health (10). This research also reveals that adolescents who have regular mealtime interactions tend to exhibit superior academic performance and a lower incidence of behavioral problems.

The structured framework provided by family meals could strengthen social values and norms, which are essential elements during the formative period of adolescence (6). Likewise, these social interactions around food may enhance the sense of belonging and identity of young people, which could impact their PA and NA (11). Furthermore, a relationship has been established between the frequency of family meals and the reduction in depressive symptoms, as well as suicidal behaviors, including thoughts, ideation and planning (12). However, the association between sharing meals with family and psychosocial wellbeing may be conditioned by the individual’s subjective perception of these interactions (13). In cases where participation in family meals does not respond to a voluntary choice on the part of the adolescent, this practice could be counterproductive and even detrimental; in such circumstances, it could undermine family or social dynamics, hindering the establishment of optimal wellbeing (14).

Social eating behavior (SEB) is distinct from family meals and focuses primarily on the enjoyment and shared experience of eating with friends or family (11). While related, SEB is broader, encompassing interactions with family, friends, and even media influences (8). It could play a significant role in shaping the eating habits of children and adolescents by fostering specific connections, feelings, and emotions related to food and body image (13). SEB emerges from a relationship of bodily, mental, and social factors that dictate when, what, and how much individuals eat (14). Adolescents tend to follow perceived social norms regarding diet, which may impact their emotional wellbeing. For example, young people might associate certain foods with PA or NA, leading to anxiety or avoidance behaviors (15). Therefore, social interaction during mealtimes could be used to promote healthier eating habits and improve mental wellbeing, as adolescents tend to adjust their food choices according to their social environment (16, 17). Moreover, one study suggested that adolescents often select foods on the basis of the image they wish to convey and align with group norms and friendships (14). Therefore, SEB, along with an individuals’ PA and NA states, may shape adolescent eating behaviors by creating associations and feelings about both food and body image (15).

Research has suggested the relevance of multiple social, economic, environmental and cultural factors in understanding the relationships between PA and NA with various mental health complications. Furthermore, previous studies have examined issues related to family meals and SEB with PA and NA, such as anxiety symptoms and eating disorders resulting from the dynamics of eating in the company of different social groups and the possible psychological effects these produce.

Studies indicate the relevance of multiple social, economic, environmental, and cultural influences could play a key role in explaining how PA and NA are linked to various mental health issues and wellbeing (18, 19). Moreover, investigations have explored family meals and SEB in relation to PA and NA with depression and anxiety symptoms and eating disorders that may emerge from the social dynamics of sharing meals with different groups and the psychological consequences they could produce (9, 13, 17). For these reasons, investigating the habits and behaviors of adolescents toward family and social meals concerning PA and NA may be relevant. Consequently, the aim of this study was to analyze the associations of family meals and SEB with PA and NA in Spanish adolescents.

## Materials and methods

This research was based on a secondary part of the data within the protocol of the Eating Healthy and Daily Life Activities (EHDLA) study, following the parameters and methodology proposed by López–Gil (20). This secondary cross-sectional study included 637 adolescents, 43% of whom were boys. The participants of the EHDLA study were adolescents aged from the *Valle de Ricote*, in the Region of Murcia, Spain, who attended three secondary schools in the area during the 2021–2022 academic year. To be part of the protocol, adolescents had to meet specific inclusion and exclusion criteria. For the participants included in the research, the parents or legal guardians of the adolescents signed an informed consent form, and the adolescents themselves also agreed to participate. It was established that adolescents between 12 and 17 years of age also reside and study in an educational center in *Valle de Ricote*. Participants who did not attend physical education classes consistently or fully were not part of the study, since the evaluations and questionnaires were conducted during these class hours. Adolescents with medical conditions that limited their physical activity or who were under medical treatment and those whose parents or guardians had not given their consent were excluded from the protocol.

The sampling frame comprised all 1496 students enrolled in three secondary schools during the 2021/2022 academic year. Of these, 1378 adolescents agreed to participate (participation rate = 92.1%). The participating schools were *Cooperativa de enseñanza (CE) El Ope* (*n* = 255; 18.5%), *Instituto de Educación Secundaria (IES) Vicente Medina* (*n* = 777; 56.4%), and *IES Pedro Guillén* (*n* = 346; 25.1%). Of the 1,378 adolescents who consented and provided baseline data, 630 (45.7%) had complete information on negative affect. After additionally excluding participants with missing data on family meals (*n* = 68; 4.9%), body mass index (BMI, *n* = 36; 2.6%), and physical activity (*n* = 7; 0.5%), the final analytic sample comprised 637 adolescents (46.2% of those who initially participated).

### Variables

#### Positive and negative affect

To measure PA and NA, the Spanish version of the Positive and Negative Affect Schedule for Children (PANAS-C10), a validated tool for assessing mood in young people, was used (21, 22). The PANAS-C10 includes 10 items: five related to negative feelings (mad, miserable, scared, sad, afraid) and five related to positive feelings (happy, proud, joyful, lively, cheerful). Participants, specifically adolescents, will rate how much they have experienced each emotion over the last few weeks. They will use a five-point Likert-type scale, where 1 signifies “very slightly or not at all” and 5 indicates “extremely.” The scores for the positive and negative items are calculated independently, creating two distinct subscale totals (21). A higher score on either the positive or negative scale reflects a stronger presence of that particular type of affect. Internal consistency of the PANAS-C10 was examined using Cronbach’s alpha (α) [with 95% confidence intervals (CIs) based on classical methods] and McDonald’s omega (ω). The PA subscale showed excellent reliability (Cronbach’s α = 0.934, 95% CI 0.925 to 0.942; McDonald’s ω = 0.937, 95% CI 0.929 to 0.944). The NA subscale demonstrated good reliability (Cronbach’s α = 0.845, 95% CI 0.825 to 0.862; McDonald’s ω = 0.852, 95% CI 0.834 to 0.870).

#### Family meals

To evaluate how often family meals occurred (frequency), participants were asked to report how many times in the previous week they had eaten with most of their household family members. The exact question was “During the past 7 days, how many times did all, or most, of your family members living in your house eat a meal together?” Responses were given on an ordinal scale with options from (a) none, (b) 1 day, (c) 2 days, (d) 3 days, (e) 4 days, (f) 5 days, (g) 6 days and (h) 7 days (23). For data collection, meals were categorized into breakfast, lunch, and dinner.

#### Social eating behavior

SEB was assessed via a self-report instrument that consists of three items designed to capture the frequency and importance of eating with others: “I like to sit down to eat with family or friends,” “Having at least one meal a day with other people (family or friends) is important to me,” and “I usually eat dinner with other people”. The participants rated their agreement with each statement via a four-point Likert scale, anchored by “strongly disagree” (1) and “strongly agree” (4). The intermediate options were “somewhat disagree” (2) and “somewhat agree” (3). A total SEB score was computed by summing the responses to the three items, yielding a possible range of 3–12 points. Higher scores reflected more frequent engagement in social eating. The reliability of this scale was supported by a Cronbach’s α of 0.70, as reported in the Eating and Activity over Time Project (24). In the EHDLA study, the SEB scale showed good internal consistency (Cronbach’s α = 0.876, 95% CI 0.861 to 0.889; McDonald’s ω = 0.881, 95% CI 0.869 to 0.894). Corrected item–total correlations ranged from 0.659 to 0.835, supporting the adequacy of each item. A one-factor solution accounted for 72.3% of the variance, with factor loadings ranging from 0.689 to 0.953, justifying the use of a summed score.

### Covariates

#### Sociodemographic factors

The participants reported their sex and age. Socioeconomic status was assessed via the Family Affluence Scale (FAS-III) (25). This tool generates a cumulative score ranging from 0 to 13 points.

#### Lifestyle factors

Physical activity and sedentary behavior were assessed via the Youth Activity Profile (YAP), a self-administered questionnaire with 15 items (26). The Spanish version of the Youth Activity Profile Questionnaire (YAP-S) was employed (27). This tool contains 15 questions divided into three sections: (1) school-related activity, (2) out-of-school activity, and (3) sedentary habits (28). Each item uses a five-point Likert scale. Sleep duration was evaluated by separately asking about weekdays and weekends: “What time do you usually go to bed?” and “What time do you usually wake up?”. The average daily sleep duration was calculated via the following formula: [(weekday sleep duration × 5) + (weekend sleep duration × 2)]/7. Energy intake was estimated through a self-administered food frequency questionnaire (FFQ), which was previously validated for the Spanish population (29).

#### Anthropometric measurements

Adolescents’ body weight was assessed via an electronic scale accurate to 0.1 kg (Tanita BC-545, Tokyo, Japan), with participants wearing light clothing. Height was measured with a portable stadiometer accurate to 0.1 cm (Leicester Tanita HR 001, Tokyo, Japan). BMI was calculated by dividing weight in kilograms by height in meters squared.

### Statistical analysis

Analyses were conducted in R (v. 4.3.2; R Core Team, Vienna, Austria) via RStudio (2023.09.1 + 494; Posit, Boston, MA, United States), with statistical significance set at a *p*-value of < 0.05. To evaluate variable distributions, the Shapiro–Wilk test was applied, complemented by density and Q–Q plots. Continuous data are presented as medians and interquartile ranges (IQRs), and categorical data are presented as percentages. As no interaction between sex and either family meal frequency (*p* = 0.764) or SEB (*p* = 0.503) was detected, analyses combined boys and girls. All primary analyses were conducted using listwise deletion, whereby participants with missing data on any of the variables included in the models were excluded. Consequently, analyses were performed on the complete cases available for all model variables (i.e., 637 participants). Associations between family meal or SEB and PA and NA were estimated via robust linear regression models fitted with the “*lmrob*()” function from the “*robustbase*” R package (30). We employed the SMDM algorithm (initial S-estimate, M-estimate, design-adaptive scale estimate, and final M-step) to accommodate heteroscedasticity and outliers. Models assumed a Gaussian family with identity link. The Tukey biweight (bisquare) psi (ψ) function with tuning constant set by the “*KS2014*” option was used for robustness. From these models, we obtained the unstandardized regression coefficients (*B*) with 95% CIs, *p*-values, and standardized beta coefficients (β). The estimated marginal means (M) and 95% CIs for the PANAS-C10 scores were derived according to the levels of family meals and SEB. Models adjusted for age, sex, socioeconomic status, physical activity, sedentary behavior, sleep duration, body mass index, and energy intake. Multicollinearity was assessed using Spearman’s rho (ρ) correlation between family meals and SEB and variance inflation factors (VIFs) for all predictors.

To account for the potential influence of missing data, we performed multiple imputation by chained equations (MICE) using the “*mice*” package. In line with recommended practice, we created 54 imputed datasets, exceeding 100 the maximum percentage of missingness in any given variable. To evaluate the plausibility of the missing at random (MAR) assumption, Little’s missing completely at random (MCAR) test was applied via the “*mcar_test*” function in the “*naniar*” package. Results indicated that the data were not missing completely at random (MCAR) [chi-square (χ^2^) = 1,255, degrees of freedom (*df*) = 238, *p* < 0.001]. Nevertheless, descriptive comparisons of participants with and without missing data on key variables showed no meaningful differences (see Supplementary Table 1), which supports the plausibility of the MAR assumption and the appropriateness of the imputation strategy.

As further sensitivity analyses, we re-estimated models using ordinary least squares (OLS) instead of robust regression, excluded energy intake from the covariate set, and rescaled energy intake to 500 kcal increments (instead of 1000 kcal as in the main analyses).

## Results

Table 1 shows the descriptive data of the study participants. The median number of weekly family meals, SEB score and PANAS-C10 scores (i.e., PA, NA) for the adolescents were 14.0 (IQR 10.0, 16.0), 10.0 (IQR 9.0, 11.0), 19.0 (IQR 15.0, 22.0), and 8.0 (IQR 6.0, 13.0), respectively.

**TABLE 1:** Descriptive data of the study participants.

Variable	N = 637^1^
Age (years)	14.0 (13.0, 15.0)
Sex	
Boys	275 (43%)
Girls	362 (57%)
FAS-III (score)	8.0 (7.0, 9.0)
Overall sleep duration (hours)	8.4 (7.6, 8.9)
YAP-S physical activity (score)	2.6 (2.2, 3.1)
YAP-S sedentary behaviors (score)	2.6 (2.2, 3.0)
Energy intake (kcal)	2589.0 (1960.0, 3443.3)
BMI (kg/m^2^)	21.7 (19.3, 25.2)
Weekly family meals (number)	14.0 (10.0, 16.0)
SEB (score)	10.0 (9.0, 11.0)
PA (score)	19.0 (15.0, 22.0)
NA (score)	8.0 (6.0, 13.0)

^1^Data expressed as median (interquartile range: 25^th^ percentile, 75^th^ percentile) or number (percentage). BMI, body mass index; FAS-III, Family Affluence Scale-III; NA, negative affect; PA, positive affect; SEB, social eating behavior; YAP-S, Spanish Youth Activity Profile.

**FIGURE 1:**
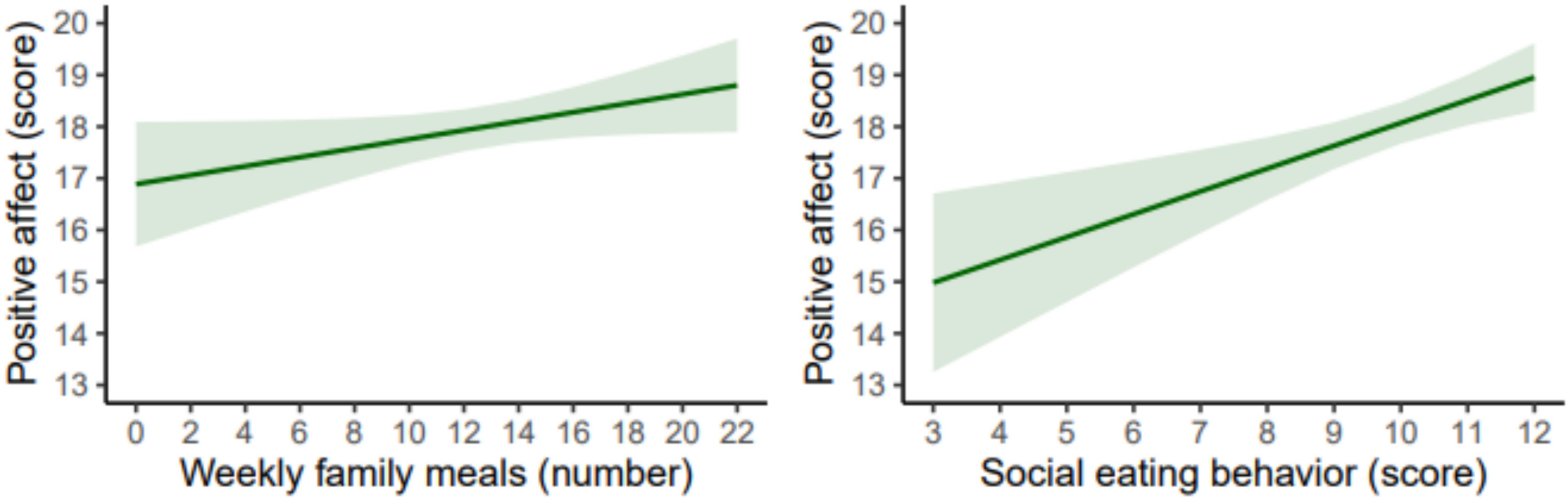
Estimated marginal means of the Positive and Negative Affect Schedule for Children score based on the number of family meals or the social eating behavior (SEB) scores in adolescents. The models were adjusted for age, sex, socioeconomic status, physical activity, sedentary behavior, sleep duration, body mass index, and energy intake.

Figure 1 shows the estimated marginal means of the PANAS-C10 (i.e., PA) according to the number of family meals and SEB scores in adolescents. For each additional family meal, the association was *B* = 0.09 (95% CI −0.001 to 0.18, *p* = 0.054; β = 0.08), while for each additional point on the SEB scale, the association was *B* = 0.44 (95% CI 0.20 to 0.69, *p* < 0.001; β = 0.15). The complete regression models corresponding to the figures are presented in Supplementary Table 2.

**FIGURE 2:**
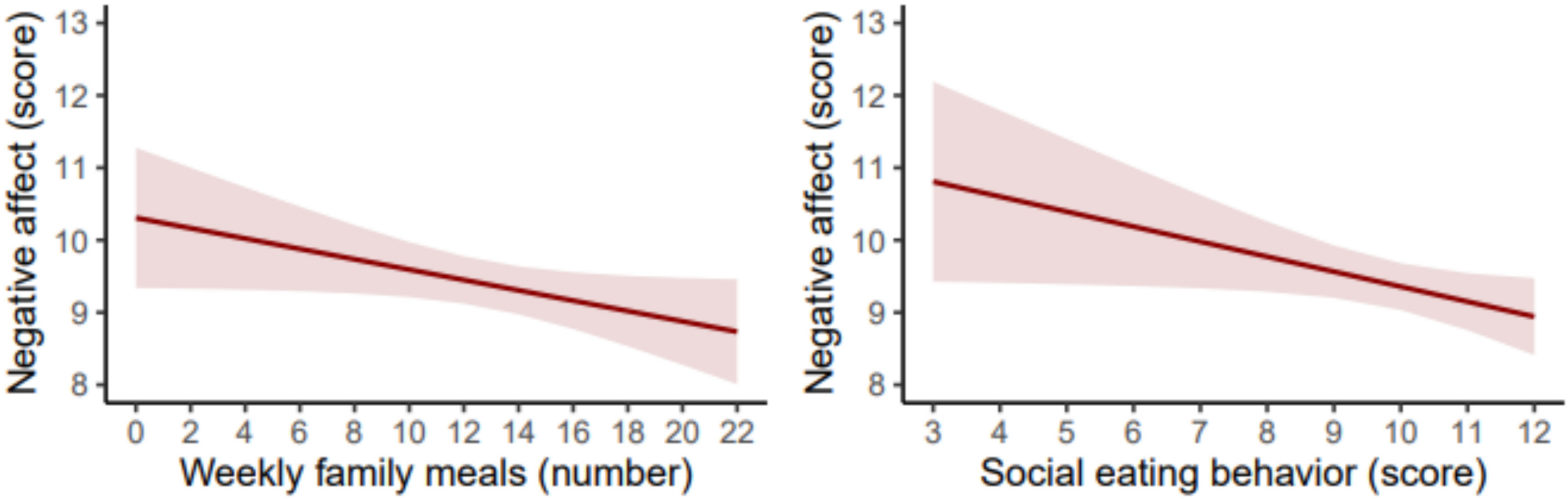
Estimated marginal means of the Positive and Negative Affect Schedule for Children score based on the number of family meals or the social eating behavior score in adolescents. The models were adjusted for age, sex, socioeconomic status, physical activity, sedentary behavior, sleep duration, body mass index, and energy intake.

Figure 2 shows the estimated marginal means of PANAS-C10 (i.e., NA) according to the number of family meals and SEB scores in adolescents. For each additional family meal, the association was *B* = –0.07 (95% CI −0.14 to −0.001, *p* = 0.048; β = −0.08), while for each additional point on the SEB scale, the association was *B* = –0.21 (95% CI −0.40 to −0.01, *p* = 0.038; β = −0.08). The complete models of the figures are shown in the Supplementary Table 3.

As part of model diagnostics, the correlation between family meals and SEB was low (Spearman’s ρ = 0.26, *p* < 0.001). VIFs ranged from 1.03 to 1.21 in the PA model and from 1.03 to 1.20 in the NA model, indicating no relevant concerns with multicollinearity. In addition, sensitivity analyses yielded results consistent with the main findings. Specifically, conclusions did not change (i) when using multiple imputation to address missing data (Supplementary Tables 4, 5), (ii) when recalculating the SEB score after removing the item “I usually eat dinner with other people” (Supplementary Tables 6, 7), (iii) when using OLS instead of robust regression (Supplementary Tables 8, 9), (iv) when rescaling energy intake to 500 kcal increments instead of 1000 kcal (Supplementary Tables 10, 11), or (v) when excluding energy intake from the models (Supplementary Tables 12, 13).

## Discussion

Our results indicate that higher SEB scores are related to greater PA in the adolescent population, whereas no association was found between family meals and PA. On the other hand, our findings suggest that higher SEB scores and a greater number of family meals are related to lower NA in adolescents.

Elgar et al. (17) reported that adolescents who shared a meal with family members and maintained adequate communication presented lower symptoms of depression, anxiety and risk behaviors, in addition to emotional wellbeing (e.g., energy, enthusiasm, and pleasurable engagement with the environment). Our results are related to these findings, as they present a potential relationship with adolescents’ mental health (31). In terms of family meals and PA, unlike our results, the study by Utter et al. (7) suggests that adolescents who more frequently shared meals with their family members presented higher wellbeing scores. However, the frequency of family meals may not reflect all the qualitative aspects of shared meals (e.g., enjoyment or quality of communication), which could help explain the difference between the findings, as they may not always be enjoyable but rather mandatory.

With respect to the relationship between SEB and NA, Victoria-Montesinos et al. (13) reported that adolescents with higher SEB scores presented lower levels of depression, anxiety and stress symptoms. This finding could be associated with our results, insofar as enjoying meals in company could be associated with reducing the possibility of facing emotionally distressing situations at specific times that are characteristic of NA. Similarly, this study (13) analyzed the associations between family meals and symptoms of depression, anxiety and stress in adolescents. The research reported that they were inversely related but that this association was not statistically significant, contrary to our findings. In the case of our results, a greater number of family meals was associated with lower levels of NA in adolescents. However, this could not establish a causal relationship.

The possible explanation for our results may be that, on occasion, family meals could be imposed. Resulting in the possibility that young people follow the rules without agreeing with them, rather than enjoying the moment, they do so to please their parents or relatives (32). This could be counterproductive to fostering a perception of belonging to the family group, which may result in an unsatisfactory feeling about the situation (32). Moreover, when attendance is obligatory or authoritarian, these occasions could become distressing rather than enjoyable for adolescents (32). In a cross-cultural investigation conducted in four European nations, was evaluated the association of shared family meals on adolescents’ dietary behaviors and self-regulatory processes. The findings suggested that, in some cultural contexts, communal meals may serve to reinforce emotional bonds and support, whereas in others, they could provoke conflict and stress (33). Nonetheless, growing evidence suggests that the family environment is critical to the development of healthy mental wellbeing in children and adolescents (34).

In the case of SEB, a possible explanation for our results is that sharing meals with friends, family, or other social groups could foster a sense of belonging and immediate enjoyment (11). Consistent with this, adolescents who regularly eat together report a stronger sense of belonging, which is a key human need linked to wellbeing and mental health (6, 35). Moreover, feeling supported and at ease with family or peers during shared meals may help reduce depressive symptoms and discourage engagement in risky behaviors (36). This finding indicates that adolescents who report greater SEB are likely to have more opportunities to voice their experiences and alleviate psychological burdens, such as distress, anxiety, or disordered eating, especially within supportive social contexts (37). Conversely, those with lower SEB may derive fewer emotional advantages from eating alongside friends or family. Shared meals could facilitate conversation that helps relieve psychological tension and intrusive thoughts (e.g., sadness, fear, guilt, and anger) (38). This process closely relates PA and NA to the dynamics present at mealtime in different contexts. This suggests that the relevant factor is the enjoyment of social interaction during meals, not only the act of eating together but also the pleasure derived from it. Such positive mealtime experiences could enhance social engagement and support adolescents’ overall wellbeing (38). Supportive social eating environments may help the presence of PA in adolescents, whereas negative mealtime contexts could exacerbate NA, thereby enhancing social enjoyment of meals by improving emotional regulation and may decrease NA (39).

The present study has several limitations. First, its cross-sectional design precludes causal inference, and longitudinal research is needed to determine whether a greater frequency of family meals and SEB directly influence PA and NA in adolescents. Second, reliance on self-reported measures introduces social desirability and recall biases in reporting both family meal frequency and SEB. Furthermore, in our study we did not evaluate possible residual confounding factors such as family functioning, parental education, and subsequent mood of adolescents, which could lead to different results. Nonetheless, this investigation also has notable strengths. To our knowledge, the relationships between family meals and SEB with respect to PA and NA in adolescents have not been previously linked in other studies. These findings provide cross-sectional evidence on the role of these dietary and social factors concerning PA and NA in a less well-studied population, such as adolescents. Furthermore, our adjusted models, including sociodemographic, anthropometric and lifestyle variables, contributed to the robustness of the results. In addition, this research was carried out with a large sample of Spanish adolescents, and this approach enabled us to obtain enough statistical power. Lastly, data collection took place shortly after the Coronavirus Disease 2019 (COVID-19) lockdown period, when students had already resumed normal daily activities. Although participants were attending school and engaging in their usual routines, we cannot fully rule out that the recent pandemic context may have influenced family mealtime patterns.

## Conclusion

Our results suggest a relationship between SEB and PA, while finding no significant association between family meals and PA, and an inverse relationship between both family meals and SEB with NA. Owing to the implications of PA and NA in the development of various physical, mental and social areas during adolescence, it is important to emphasize the possible protective role of healthy family and social relationships. Participation in family meals and the promotion of positive social eating environments could help promote PA and may reduce the prevalence of NA and its negative consequences. Future research should highlight the importance of fostering favorable environments and encouraging healthy behaviors to mitigate NA and its apparent associated risks.

## Data Availability

The raw data supporting the conclusions of this article will be made available by the authors, without undue reservation.
