# Supplementary material for: Associations between social eating contexts and affective states in adolescents: the EHDLA study
Source: Front Nutr. 2025 Oct 15;12:1653965. doi: 10.3389/fnut.2025.1653965 (PMC12568017; doi:10.3389/fnut.2025.1653965)
Supplement: Supplementary file 1 [file Supplementary_file_1.docx]

**Supplementary material**

**Table S1.** Comparison of descriptive data of participants between listwise deletion method and multiple imputation method.

| **Variable** | **Listwise deletion method** | **Multiple imputation method** |
| --- | --- | --- |
| Age | 14.0 (13.0, 16.0) | 14.0 (13.0, 16.0) |
| Sex |  |  |
| Boys | 680 (49.3%) | 680 (49.3%) |
| Girls | 698 (50.7%) | 698 (50.7%) |
| FAS-III (score) | 8.0 (7.0, 10.0) | 8.0 (7.0, 10.0) |
| Missing | 267 | 0 |
| Overall sleep duration (hours) | 8.2 (7.6, 8.8) | 8.2 (7.5, 8.8) |
| Missing | 277 | 0 |
| YAP-S physical activity (score) | 2.6 (2.2, 3.1) | 2.6 (2.1, 3.0) |
| Missing | 296 | 0 |
| YAP-S sedentary behaviors (score) | 2.6 (2.2, 3.0) | 2.6 (2.2, 3.0) |
| Missing | 296 | 0 |
| Energy intake (kcal) | 2590.5 (1959.3, 3472.9) | 2623.0 (1978.1, 3646.1) |
| Missing | 442 | 0 |
| BMI (kg/m^2^) | 21.7 (19.3, 25.4) | 21.7 (19.4, 25.4) |
| Missing | 117 | 0 |
| Weekly family meals (number) | 14.0 (10.0, 16.0) | 13.0 (9.0, 16.0) |
| Missing | 471 | 0 |
| SEB (score) | 10.0 (8.0, 11.0) | 10.0 (8.0, 11.0) |
| Missing | 383 | 0 |
| PA (score) | 18.0 (14.0, 22.0) | 18.0 (14.0, 21.0) |
| Missing | 630 | 0 |
| NA (score) | 9.0 (6.0, 13.0) | 9.0 (6.0, 13.0) |
| Missing | 630 | 0 |
| BMI, body mass index; FAS-III, Family Affluence Scale-III; NA, negative affect; PA, positive affect; SEB, social eating behavior; YAP-S, Spanish Youth Activity Profile. | | |

Table S2. Robust generalized linear model examining the associations of family meals and SEB (and covariates) with PA in adolescents (analytic N = 637).

| **Predictors** | ***B*** | **95% CI** | ***p* value** | *β* |
| --- | --- | --- | --- | --- |

| Weekly family meals (per meal) | 0.09 | -0.001, 0.18 | 0.054 | 0.08 |
| --- | --- | --- | --- | --- |
| Social eating behavior (per point) | 0.44 | 0.20, 0.69 | <0.001 | 0.15 |
| Age (per year) | -0.11 | -0.39, 0.17 | 0.437 | -0.03 |
| Sex |  |  |  |  |
| Boys | Reference |  |  |  |
| Girls | -2.15 | -3.00, -1.30 | <0.001 | -0.21 |
| FAS-III (per point) | 0.23 | 0.04, 0.43 | 0.019 | 0.09 |
| Overall sleep duration (per hour) | 0.57 | 0.09, 1.06 | 0.021 | 0.10 |
| YAP-S physical activity (per point) | 0.37 | -0.24, 0.98 | 0.235 | 0.05 |
| YAP-S sedentary behaviors (per point) | 0.34 | -0.36, 1.05 | 0.337 | 0.04 |
| Energy intake (per 1000 kcal) | -0.03 | -0.23, 0.17 | 0.738 | -0.01 |
| BMI (per kg/m^2^) | -0.06 | -0.15, 0.02 | 0.152 | -0.06 |

| *β*, standardized beta coefficient; *B*, unstandardized beta coefficient; BMI, body mass index; CI, confidence interval; FAS-III, Family Affluence Scale-III; PA, positive affect; SEB, social eating behavior; YAP-S, Spanish Youth Activity Profile. Note: For energy intake, the coefficient is expressed per 1000 kcal to facilitate interpretation, as reporting changes per 1 kcal would yield extremely small beta coefficients. |
| --- |

Table S3. Robust generalized linear model examining the associations of family meals and SEB (and covariates) with NA in adolescents (analytic N = 637).

| **Predictors** | ***B*** | **95% CI** | ***p* value** | *β* |
| --- | --- | --- | --- | --- |
| Weekly family meals (per meal) | -0.07 | -0.14, -0.001 | 0.048 | -0.08 |
| Social eating behavior (per point) | -0.21 | -0.40, -0.01 | 0.038 | -0.08 |
| Age (per year) | -0.04 | -0.26, 0.19 | 0.753 | -0.01 |
| Sex |  |  |  |  |
| Boys | Reference |  |  |  |
| Girls | 2.45 | 1.77, 3.14 | <0.001 | 0.27 |
| FAS-III (per point) | -0.17 | -0.33, -0.01 | 0.035 | -0.08 |
| Overall sleep duration (per hour) | -0.39 | -0.79, -0.003 | 0.049 | -0.08 |
| YAP-S physical activity (per point) | -0.14 | -0.64, 0.35 | 0.571 | -0.02 |
| YAP-S sedentary behaviors (per point) | 0.22 | -0.35, 0.79 | 0.441 | 0.03 |
| Energy intake (per 1000 kcal) | -0.001 | -0.17, 0.17 | 0.983 | -0.001 |
| BMI (per kg/m^2^) | 0.01 | -0.06, 0.07 | 0.887 | 0.01 |
| *β*, standardized beta coefficient; *B*, unstandardized beta coefficient; BMI, body mass index; CI, confidence interval; FAS-III, Family Affluence Scale-III; NA, negative affect. SEB, social eating behavior; YAP-S, Spanish Youth Activity Profile. Note: For energy intake, the coefficient is expressed per 1000 kcal to facilitate interpretation, as reporting changes per 1 kcal would yield extremely small beta coefficients. | | | | |

Table S4. Robust generalized linear model examining the associations of family meals and social eating behavior (and covariates) with positive affect in adolescents, using a multiple imputation method (analytic N = 1378).

| **Predictors** | ***B*** | **95% CI** | ***p* value** | *β* |
| --- | --- | --- | --- | --- |
| Weekly family meals (per meal) | 0.12 | 0.06, 0.18 | <0.001 | 0.15 |
| Social eating behavior (per point) | 0.25 | 0.15, 0.34 | <0.001 | 0.11 |
| Age (per year) | -0.16 | -0.33, 0.01 | 0.066 | -0.06 |
| Sex |  |  |  |  |
| Boys | Reference |  |  |  |
| Girls | -2.26 | -2.83, -1.69 | <0.001 | -0.27 |
| FAS-III (per point) | 0.18 | 0.05, 0.30 | 0.006 | 0.10 |
| Overall sleep duration (per hour) | 0.86 | 0.55, 1.17 | <0.001 | 0.08 |
| YAP-S physical activity (per point) | -0.30 | -0.72, 0.11 | 0.152 | -0.01 |
| YAP-S sedentary behaviors (per point) | 0.33 | -0.13, 0.80 | 0.163 | 0.03 |
| Energy intake (per 1000 kcal) | 0.05 | -0.06, 0.17 | 0.370 | -0.01 |
| BMI (per kg/m^2^) | -0.07 | -0.12, -0.01 | 0.023 | -0.14 |
| *β*, standardized beta coefficient; *B*, unstandardized beta coefficient; BMI, body mass index; CI, confidence interval; FAS-III, Family Affluence Scale-III; PA, positive affect; SEB, social eating behavior; YAP-S, Spanish Youth Activity Profile. Note: For energy intake, the coefficient is expressed per 1000 kcal to facilitate interpretation, as reporting changes per 1 kcal would yield extremely small beta coefficients. | | | | |

Table S5. Robust generalized linear model examining the associations of family meals and social eating behavior (and covariates) with negative affect in adolescents, using a multiple imputation method (analytic N = 1378).

| **Predictors** | ***B*** | **95% CI** | ***p* value** | *β* |
| --- | --- | --- | --- | --- |

| Weekly family meals (per meal) | -0.10 | -0.14, -0.05 | <0.001 | -0.11 |
| --- | --- | --- | --- | --- |
| Social eating behavior (per point) ^†^ | -0.12 | -0.19, -0.04 | 0.002 | -0.08 |
| Age (per year) | -0.01 | -0.14, 0.13 | 0.936 | -0.02 |
| Sex |  |  |  |  |
| Boys | Reference |  |  |  |
| Girls | 2.36 | 1.91, 2.81 | <0.001 | 0.26 |
| FAS-III (per point) | -0.13 | -0.23, -0.03 | 0.011 | -0.06 |
| Overall sleep duration (per hour) | -0.47 | -0.72, -0.23 | <0.001 | -0.10 |
| YAP-S physical activity (per point) | 0.07 | -0.26, 0.40 | 0.671 | 0.01 |
| YAP-S sedentary behaviors (per point) | 0.02 | -0.34, 0.39 | 0.900 | 0.003 |
| Energy intake (per 1000 kcal) | -0.03 | -0.13, 0.06 | 0.483 | -0.02 |
| BMI (per kg/m^2^) | -0.01 | -0.06, 0.03 | 0.524 | -0.02 |
| *β*, standardized beta coefficient; *B*, unstandardized beta coefficient; BMI, body mass index; CI, confidence interval; FAS-III, Family Affluence Scale-III; PA, positive affect; SEB, social eating behavior; YAP-S, Spanish Youth Activity Profile. ^†^ SEB score was recalculated after removing the item “I usually eat dinner with other people”, due to its conceptual overlap with family meals. Note: For energy intake, the coefficient is expressed per 1000 kcal to facilitate interpretation, as reporting changes per 1 kcal would yield extremely small beta coefficients. | | | | |

Table S6. Sensitivity analysis with a robust generalized linear model examining the associations of family meals and social eating behavior, adjusted for covariates, with positive affect in adolescents (analytic N = 637).

| **Predictors** | ***B*** | **95% CI** | ***p* value** | *β* |
| --- | --- | --- | --- | --- |
| Weekly family meals (per meal) | -0.05 | -0.12, 0.02 | 0.147 | 0.07 |
| Social eating behavior (per point) ^†^ | -0.61 | -0.89, -0.33 | <0.001 | 0.21 |
| Age (per year) | -0.04 | -0.26, 0.18 | 0.711 | -0.03 |
| Sex |  |  |  |  |
| Boys | Reference |  |  |  |
| Girls | 2.48 | 1.80, 3.15 | <0.001 | -0.21 |
| FAS-III (per point) | -0.17 | -0.32, -0.01 | 0.038 | 0.10 |
| Overall sleep duration (per hour) | -0.34 | -0.73, 0.05 | 0.087 | 0.10 |
| YAP-S physical activity (per point) | -0.13 | -0.61, 0.36 | 0.614 | 0.04 |
| YAP-S sedentary behaviors (per point) | 0.18 | -0.39, 0.74 | 0.539 | 0.05 |
| Energy intake (per 1000 kcal) | -0.02 | -0.20, 0.15 | 0.791 | -0.01 |
| BMI (per kg/m^2^) | 0.00 | -0.06, 0.07 | 0.893 | -0.06 |
| *β*, standardized beta coefficient; *B*, unstandardized beta coefficient; BMI, body mass index; CI, confidence interval; FAS-III, Family Affluence Scale-III; PA, positive affect; SEB, social eating behavior; YAP-S, Spanish Youth Activity Profile. ^†^ SEB score was recalculated after removing the item “I usually eat dinner with other people”, due to its conceptual overlap with family meals. Note: For energy intake, the coefficient is expressed per 1000 kcal to facilitate interpretation, as reporting changes per 1 kcal would yield extremely small beta coefficients. | | | | |

Table S7. Sensitivity analysis with a robust generalized linear model examining the associations of family meals and social eating behavior, adjusted for covariates, with negative affect in adolescents (analytic N = 637).

| **Predictors** | ***B*** | **95% CI** | ***p* value** | *β* |
| --- | --- | --- | --- | --- |

| Weekly family meals (per meal) | 0.07 | -0.01, 0.16 | 0.098 | -0.06 |
| --- | --- | --- | --- | --- |
| Social eating behavior (per point) ^†^ | 0.87 | 0.52, 1.21 | <0.001 | -0.17 |
| Age (per year) | -0.11 | -0.39, 0.16 | 0.415 | -0.01 |
| Sex |  |  |  |  |
| Boys | Reference |  |  |  |
| Girls | -2.14 | -2.98, -1.31 | <0.001 | 0.27 |
| FAS-III (per point) | 0.25 | 0.06, 0.44 | 0.038 | -0.08 |
| Overall sleep duration (per hour) | 0.01 | 0.00, 0.02 | 0.087 | -0.07 |
| YAP-S physical activity (per point) | 0.28 | -0.33, 0.88 | 0.614 | -0.02 |
| YAP-S sedentary behaviors (per point) | 0.44 | -0.25, 1.13 | 0.539 | 0.02 |
| Energy intake (per 1000 kcal) | -0.02 | -0.22, 0.18 | 0.791 | -0.01 |
| BMI (per kg/m^2^) | -0.06 | -0.15, 0.02 | 0.893 | 0.01 |
| *β*, standardized beta coefficient; *B*, unstandardized beta coefficient; BMI, body mass index; CI, confidence interval; FAS-III, Family Affluence Scale-III; PA, positive affect; SEB, social eating behavior; YAP-S, Spanish Youth Activity Profile. ^†^ SEB score was recalculated after removing the item “I usually eat dinner with other people”, due to its conceptual overlap with family meals. Note: For energy intake, the coefficient is expressed per 1000 kcal to facilitate interpretation, as reporting changes per 1 kcal would yield extremely small beta coefficients. | | | | |

Table S8. Sensitivity analysis with a generalized linear model examining the associations of family meals and social eating behavior, adjusted for covariates, with positive affect in adolescents (analytic N = 637).

| **Predictors** | ***B*** | **95% CI** | ***p* value** | *β* |
| --- | --- | --- | --- | --- |
| Weekly family meals (per meal) | 0.08 | -0.0004, 0.17 | 0.051 | 0.08 |
| Social eating behavior (per point) | 0.37 | 0.14, 0.59 | 0.002 | 0.13 |
| Age (per year) | -0.15 | -0.42, 0.11 | 0.266 | -0.05 |
| Sex |  |  |  |  |
| Boys | Reference |  |  |  |
| Girls | -2.03 | -2.84, -1.23 | <0.001 | -0.20 |
| FAS-III (per point) | 0.23 | 0.05, 0.42 | 0.015 | 0.09 |
| Overall sleep duration (per hour) | 0.01 | 0.00, 0.02 | 0.028 | 0.09 |
| YAP-S physical activity (per point) | 0.38 | -0.20, 0.97 | 0.198 | 0.05 |
| YAP-S sedentary behaviors (per point) | 0.35 | -0.31, 1.02 | 0.299 | 0.04 |
| Energy intake (per 1000 kcal) | -0.01 | -0.20, 0.18 | 0.933 | -0.003 |
| BMI (per kg/m^2^) | -0.07 | -0.16, 0.01 | 0.076 | -0.08 |
| *β*, standardized beta coefficient; *B*, unstandardized beta coefficient; BMI, body mass index; CI, confidence interval; FAS-III, Family Affluence Scale-III; PA, positive affect; SEB, social eating behavior; YAP-S, Spanish Youth Activity Profile. Note: As a sensitivity analysis, ordinary least squares (OLS) regression was used instead of the robust regression approach applied in the main analysis. For energy intake, the coefficient is expressed per 1000 kcal to facilitate interpretation, as reporting changes per 1 kcal would yield extremely small beta coefficients. | | | | |

Table S9. Sensitivity analysis with a generalized linear model examining the associations of family meals and social eating behavior, adjusted for covariates, with negative affect in adolescents (analytic N = 637).

| **Predictors** | ***B*** | **95% CI** | ***p* value** | *β* |
| --- | --- | --- | --- | --- |
| Weekly family meals (per meal) | -0.08 | -0.15, -0.01 | 0.036 | -0.08 |
| Social eating behavior (per point) | -0.19 | -0.39, 0.01 | 0.061 | -0.08 |
| Age (per year) | -0.04 | -0.27, 0.19 | 0.758 | -0.01 |
| Sex |  |  |  |  |
| Boys | Reference |  |  |  |
| Girls | 2.57 | 1.88, 3.27 | <0.001 | 0.28 |
| FAS-III (per point) | -0.19 | -0.35, -0.02 | 0.025 | -0.09 |
| Overall sleep duration (per hour) | -0.01 | -0.01, 0.00 | 0.028 | -0.09 |
| YAP-S physical activity (per point) | -0.09 | -0.60, 0.41 | 0.719 | -0.04 |
| YAP-S sedentary behaviors (per point) | 0.15 | -0.43, 0.74 | 0.601 | 0.02 |
| Energy intake (per 1000 kcal) | 0.05 | -0.11, 0.22 | 0.532 | 0.02 |
| BMI (per kg/m^2^) | 0.02 | -0.05, 0.09 | 0.620 | 0.02 |
| *β*, standardized beta coefficient; *B*, unstandardized beta coefficient; BMI, body mass index; CI, confidence interval; FAS-III, Family Affluence Scale-III; PA, positive affect; SEB, social eating behavior; YAP-S, Spanish Youth Activity Profile. Note: As a sensitivity analysis, ordinary least squares (OLS) regression was used instead of the robust regression approach applied in the main analysis. For energy intake, the coefficient is expressed per 1000 kcal to facilitate interpretation, as reporting changes per 1 kcal would yield extremely small beta coefficients. | | | | |

Table S10. Sensitivity analysis with a robust generalized linear model examining the associations of family meals and social eating behavior, adjusted for covariates, with positive affect in adolescents (analytic N = 637).

| **Predictors** | ***B*** | **95% CI** | ***p* value** | *β* |
| --- | --- | --- | --- | --- |
| Weekly family meals (per meal) | 0.08 | 0.00, 0.17 | 0.051 | 0.08 |
| Social eating behavior (per point) | 0.37 | 0.14, 0.59 | 0.002 | 0.15 |
| Age (per year) | -0.15 | -0.42, 0.11 | 0.266 | -0.03 |
| Sex |  |  |  |  |
| Boys | Reference |  |  |  |
| Girls | -2.03 | -2.84, -1.23 | <0.001 | -0.21 |
| FAS-III (per point) | 0.23 | 0.05, 0.42 | 0.015 | 0.09 |
| Overall sleep duration (per hour) | 0.01 | 0.00, 0.02 | 0.028 | 0.10 |
| YAP-S physical activity (per point) | 0.38 | -0.20, 0.97 | 0.198 | 0.05 |
| YAP-S sedentary behaviors (per point) | 0.35 | -0.31, 1.02 | 0.299 | -0.01 |
| Energy intake (per 500 kcal) | 0.00 | 0.00, 0.00 | 0.933 | -0.001 |
| BMI (per kg/m^2^) | -0.07 | -0.16, 0.01 | 0.076 | -0.06 |
| *β*, standardized beta coefficient; *B*, unstandardized beta coefficient; BMI, body mass index; CI, confidence interval; FAS-III, Family Affluence Scale-III; PA, positive affect; SEB, social eating behavior; YAP-S, Spanish Youth Activity Profile. Note: As a sensitivity analysis, energy intake was rescaled so that the coefficient is expressed per 500 kcal increase (rather than per 1000 kcal as in the main analysis). | | | | |

Table S11. Sensitivity analysis with a robust generalized linear model examining the associations of family meals and social eating behavior, adjusted for covariates, with negative affect in adolescents (analytic N = 637).

| **Predictors** | ***B*** | **95% CI** | ***p* value** | *β* |
| --- | --- | --- | --- | --- |
| Weekly family meals (per meal) | -0.07 | -0.14, -0.001 | 0.048 | -0.08 |
| Social eating behavior (per point) | -0.21 | -0.40, -0.01 | 0.038 | -0.08 |
| Age (per year) | -0.04 | -0.26, 0.19 | 0.753 | -0.01 |
| Sex |  |  |  |  |
| Boys | Reference |  |  |  |
| Girls | 2.45 | 1.77, 3.14 | <0.001 | 0.27 |
| FAS-III (per point) | -0.17 | -0.33, -0.01 | 0.035 | -0.08 |
| Overall sleep duration (per hour) | -0.01 | -0.01, 0.00 | 0.049 | -0.08 |
| YAP-S physical activity (per point) | -0.14 | -0.64, 0.35 | 0.571 | -0.02 |
| YAP-S sedentary behaviors (per point) | 0.22 | -0.35, 0.79 | 0.441 | 0.03 |
| Energy intake (per 500 kcal) | 0.00 | -0.09, 0.09 | 0.983 | -0.001 |
| BMI (per kg/m^2^) | 0.01 | -0.06, 0.07 | 0.887 | 0.005 |
| *β*, standardized beta coefficient; *B*, unstandardized beta coefficient; BMI, body mass index; CI, confidence interval; FAS-III, Family Affluence Scale-III; NA, negative affect. SEB, social eating behavior; YAP-S, Spanish Youth Activity Profile. Note: As a sensitivity analysis, energy intake was rescaled so that the coefficient is expressed per 500 kcal increase (rather than per 1000 kcal as in the main analysis). | | | | |

Table S12. Sensitivity analysis using a robust generalized linear model examining the associations of family meals and social eating behavior (and covariates) with positive affect in adolescents (analytic N = 637).

| **Predictors** | ***B*** | **95% CI** | ***p* value** | *β* |
| --- | --- | --- | --- | --- |
| Weekly family meals (per meal) | 0.09 | -0.002, 0.17 | 0.056 | 0.08 |
| Social eating behavior (per point) | 0.44 | 0.20, 0.69 | <0.001 | 0.15 |
| Age (per year) | -0.11 | -0.39, 0.16 | 0.422 | -0.03 |
| Sex |  |  |  |  |
| Boys | Reference |  |  |  |
| Girls | -2.16 | -3.01, -1.32 | <0.001 | -0.21 |
| FAS-III (per point) | 0.24 | 0.04, 0.43 | 0.018 | 0.10 |
| Overall sleep duration (per hour) | 0.01 | 0.00, 0.02 | 0.020 | 0.10 |
| YAP-S physical activity (per point) | 0.36 | -0.25, 0.97 | 0.248 | 0.05 |
| YAP-S sedentary behaviors (per point) | 0.33 | -0.37, 1.02 | 0.355 | 0.04 |
| BMI (per kg/m^2^) | -0.06 | -0.15, 0.02 | 0.152 | -0.06 |
| *β*, standardized beta coefficient; *B*, unstandardized beta coefficient; BMI, body mass index; CI, confidence interval; FAS-III, Family Affluence Scale-III; PA, positive affect; SEB, social eating behavior; YAP-S, Spanish Youth Activity Profile. Note: As a sensitivity analysis, models were re-estimated without adjustment for energy intake. | | | | |

Table S13. Sensitivity analysis using a robust generalized linear model examining the associations of family meals and social eating behavior (and covariates) with negative affect in adolescents (analytic N = 637).

| **Predictors** | ***B*** | **95% CI** | ***p* value** | *β* |
| --- | --- | --- | --- | --- |
| Weekly family meals (per meal) | -0.07 | -0.14, -0.001 | 0.048 | -0.08 |
| Social eating behavior (per point) | -0.21 | -0.40, -0.01 | 0.038 | -0.08 |
| Age (per year) | -0.04 | -0.26, 0.19 | 0.752 | -0.01 |
| Sex |  |  |  |  |
| Boys | Reference |  |  |  |
| Girls | 2.45 | 1.77, 3.13 | <0.001 | 0.27 |
| FAS-III (per point) | -0.17 | -0.33, -0.01 | 0.034 | -0.08 |
| Overall sleep duration (per hour) | -0.01 | -0.01, 0.00 | 0.048 | -0.08 |
| YAP-S physical activity (per point) | -0.14 | -0.63, 0.35 | 0.566 | -0.02 |
| YAP-S sedentary behaviors (per point) | 0.22 | -0.34, 0.79 | 0.437 | 0.03 |
| BMI (per kg/m^2^) | 0.01 | -0.06, 0.07 | 0.886 | 0.01 |
| *β*, standardized beta coefficient; *B*, unstandardized beta coefficient; BMI, body mass index; CI, confidence interval; FAS-III, Family Affluence Scale-III; PA, positive affect; SEB, social eating behavior; YAP-S, Spanish Youth Activity Profile. Note: As a sensitivity analysis, models were re-estimated without adjustment for energy intake. | | | | |
